# Changes in Within-Shoot Carbon Partitioning in Pinot Noir Grapevines Subjected to Early Basal Leaf Removal

**DOI:** 10.3389/fpls.2018.01122

**Published:** 2018-08-03

**Authors:** Tommaso Frioni, Dana Acimovic, Sergio Tombesi, Paolo Sivilotti, Alberto Palliotti, Stefano Poni, Paolo Sabbatini

**Affiliations:** ^1^Dipartimento di Scienze delle Produzioni Vegetali Sostenibili, Università Cattolica del Sacro Cuore, Piacenza, Italy; ^2^Department of Horticulture, Michigan State University, East Lansing, MI, United States; ^3^Department of Agricultural, Food, Environmental and Animal Sciences, University of Udine, Udine, Italy; ^4^Dipartimento di Scienze Agrarie, Alimentari e Ambientali, Università di Perugia, Perugia, Italy

**Keywords:** defoliation, fruit set, carbon discrimination, source-to-sink ratio, *Vitis vinifera* L

## Abstract

Early leaf removal significantly alters the source-sink balance within grapevine shoots, leading to a reduction in fruit set. However, no research has previously examined the conditions controlling this process in terms of carbon allocation among major sink organs following defoliation. In this study, the impact of defoliation at bloom on the distribution dynamics of leaf assimilates among clusters and growing shoot apices was investigated on *Vitis vinifera*, cv. Pinot noir, grown in Michigan, a cool climate viticultural region. Three levels of defoliation: no leaves removed (LR-0); six leaves removed from six basal nodes (LR-6); and ten leaves removed from ten basal nodes (LR-10), were imposed at full bloom. A ^13^C pulsing was performed 1 week after the treatment application to the defoliated shoots. Single leaf gas exchange (P_n_), diurnal changes of the leaf net CO_2_ assimilation rate, carbon distribution, fruit-set, yield, and fruit composition were measured. Higher P_n_ was recorded in diurnal measurements of gas exchange in leaf removal (LR) treatments compared to LR-0. The shoot apex of LR-10 experienced the highest ^13^C allocation (%) after 3 and 7 days following the carbon pulsing. LR-10 had lower percentage of ^13^C allocated to clusters, which decreased fruit set by 60%, compared to the control, and enhanced the concentration of phenolic compounds in fruit. Alteration of carbon portioning among shoot sink organs indicated that an increasing severity of leaf removal significantly reduced fruit set, and was linearly correlated to shoot apex sink strength, which occurred at the expense of the cluster.

## Introduction

The source-sink balance represents an important aspect in the evolutionary strategy adopted by perennials regarding carbon allocation; reproduction is a cost for the plant because it competes with vegetative growth (Obeso, 2002). Further to its applicative importance, the management of source-sink interactions in higher plants are at the cornerstone of potential plant productivity and represent a sustainable strategy to improve production quality in many fruit crops (Flore and Lakso, 1989). In grapevines, a calibrated source-sink imbalance, generated through the removal of part of photosynthetically active leaf area, has become a key practice to regulate productivity and berry composition in commercial vineyards (Poni et al., 2006; Hed et al., 2009; Sabbatini and Howell, 2010).

Following defoliation, the drastic reduction in main shoot leaf area fosters the production of more lateral shoots and leaves and delays senescence of remaining leaves (Candolfi-Vasconcelos and Koblet, 1990). In addition, the photosynthetic efficiency of retained leaves is higher because of the decrease in secondary feedback inhibition of photosynthesis. This occurs because of the primary effect on phloem loading and unloading stimulated by the high sink demand and the low source availability (Lemoine et al., 2013). Moreover, reduced leaf area conditions, led to a higher carboxylation efficiency, as well as an enhanced capacity for regeneration of ribulose- 1.5-bisphosphate (Flore and Lakso, 1989), mesophyll thickening and an increase in chlorophyll concentration (Candolfi-Vasconcelos and Koblet, 1991; Poni et al., 2006, 2008; Palliotti et al., 2011).

Perennial and non-perennial specie, when severe defoliations occur or are artificially imposed, may undertake adaptation mechanisms that favor vegetative activity or, vice-versa, reproductive activity, depending by many factors. Annual plants, are able to mobilize carbon to produce new photosynthetic tissues if leaf curtails occur far from flowering (Castrillón-Arbeláez et al., 2012; Vargas-Ortiz et al., 2013, 2015). In those stages when reproductive activity is highly demanding for energy and carbon, this dynamics might be altered (Bennett et al., 2005; Vargas-Ortiz et al., 2013, 2015). Grapevines and other perennials in general respond similarly, but they can account on a relevant source of reserves allocated on permanent organs (Candolfi-Vasconcelos et al., 1994; Bennett et al., 2005; Galiano et al., 2011). However, different dynamics might be related to survival strategies related to the presence of permanent organs in perennial crops, whereas annual plants are strictly dependent to reproductive activity to overcome different seasons. In grapevine, it is well-understood that the source limitation induced by early leaf removal applied around bloom, promotes a reduction in fruit-set (Poni et al., 2008; Tardaguila et al., 2012; Acimovic et al., 2016). A potential explanation for this effect is related to the carbon starvation caused by the removal of the most active portion of the shoot at an early phenological stage of the grapevine growth and development (Poni et al., 2008; Palliotti et al., 2011). Due to this decrease in source availability, defoliation alters carbon assimilation patterns to important sinks in a time-dependent way. During bloom, translocation of carbon labeled photo-assimilates (^14^C) between different grapevine shoots bourn on the same vine occurred when they were severely defoliated (Quinlan and Weaver, 1970). However, several experiments carried out under varying climatic conditions have shown that the removal of leaf area has to be quite severe (i.e., 60–80% of the standing leaf area) in order to achieve a significant reduction in berry fruit set. Indeed, if early defoliation does not reach a specific intensity threshold, berry-set may be unmodified in comparison with undefoliated vines (Gatti et al., 2015; Acimovic et al., 2016). Therefore, targeted early defoliation has become a common management strategy for the control of excessive cropping in high-yielding cultivars (Poni et al., 2006) or in cultivars characterized by a compact cluster and prone to cluster rot complex at harvest (Hed et al., 2009; Sabbatini and Howell, 2010). Because of this, it is extensively used in cool climate viticulture to improve fruit quality through the reduction of cluster compactness, and subsequent decrease in bunch rot severity (Mosetti et al., 2016). Pinot Noir is one such cultivar where early removal of six leaves was used in a 3-year study with success in bunch rot control and fruit quality improvement (Acimovic et al., 2016). Specifically, defoliation has been shown to increase soluble solids, total phenolics and anthocyanin content, which leads to higher quality must composition at harvest (Poni et al., 2004, 2009; Lemut et al., 2011; Kotseridis et al., 2012; Palliotti et al., 2012; Lee and Skinkis, 2013).

Currently, no information exists to explain the effect of early (i.e., around flowering) leaf removal on carbon partitioning between primary sink organs of the growing shoot. Our hypothesis was that the nature of the defoliation effects on the reproductive activity (i.e., fruit set) involve substantial modifications of the sink prioritization in the shoot, in addition to the previously understood reduction in carbon supply. The aim of the present work was therefore to determine: (i) the patterns of carbon production and allocation to different shoot organs at different levels of early defoliation and (ii) correlations between the above patterns with physiological parameters and fruit set.

## Materials and methods

### Plant material and experimental design

The research was conducted at the Southwest Michigan Research and Extension Center (latitude 40°09′N, longitude 86°36′W, elevation 220 m) near Benton Harbor, Michigan. During 2011, data were collected in a 10-year-old vineyard of *V*itis *vinifera* L. cv. Pinot Noir (clone 777 grafted onto 3309C), with a spacing of 1.8 m between vines and 3.0 m between rows and trained to a vertical shoot positioning system. Vines were winter pruned to three-node spurs, leaving ~60 buds per vine. No additional shoot or cluster thinning was performed before application of the treatments, and the vines were carrying about 80 clusters (1.4 clusters per shoot). The pest management program was based on scouting, experience, and weather conditions. Shoots were trimmed on 14 July when they reached 30 cm above the highest pair of catch wires (2.1 m). The vineyard was rain fed and pertinent temperature data were recorded during the experiment by an automated weather station from the Michigan Automated Weather Network (MAWN) located on the site at 120 m from the experimental vineyard. Total monthly precipitation, daily precipitation, daily minimum, maximum, and average temperature and Growing Degree Days (GDD) calculated with the Baskerville-Emin method using a base temperature of 10°C (Baskerville and Emin, 1969). The lowest daily temperature of 2.8°C was recorded on May 16 and it was not harmful to young developing shoots. The maximum daily temperature of 35.5°C was recorded on July 20, which coincided with a lag phase of berry development. The seasonal growing degree accumulation was 1467 GDD and the total precipitation for entire growing season was 592 mm. During the experimental period, rain occurred approximately twice per week for a total of 62.2 mm and mean temperature fluctuated between 15 and 25°C, providing the optimum conditions for flowering and fruit set.

**Figure 1:**
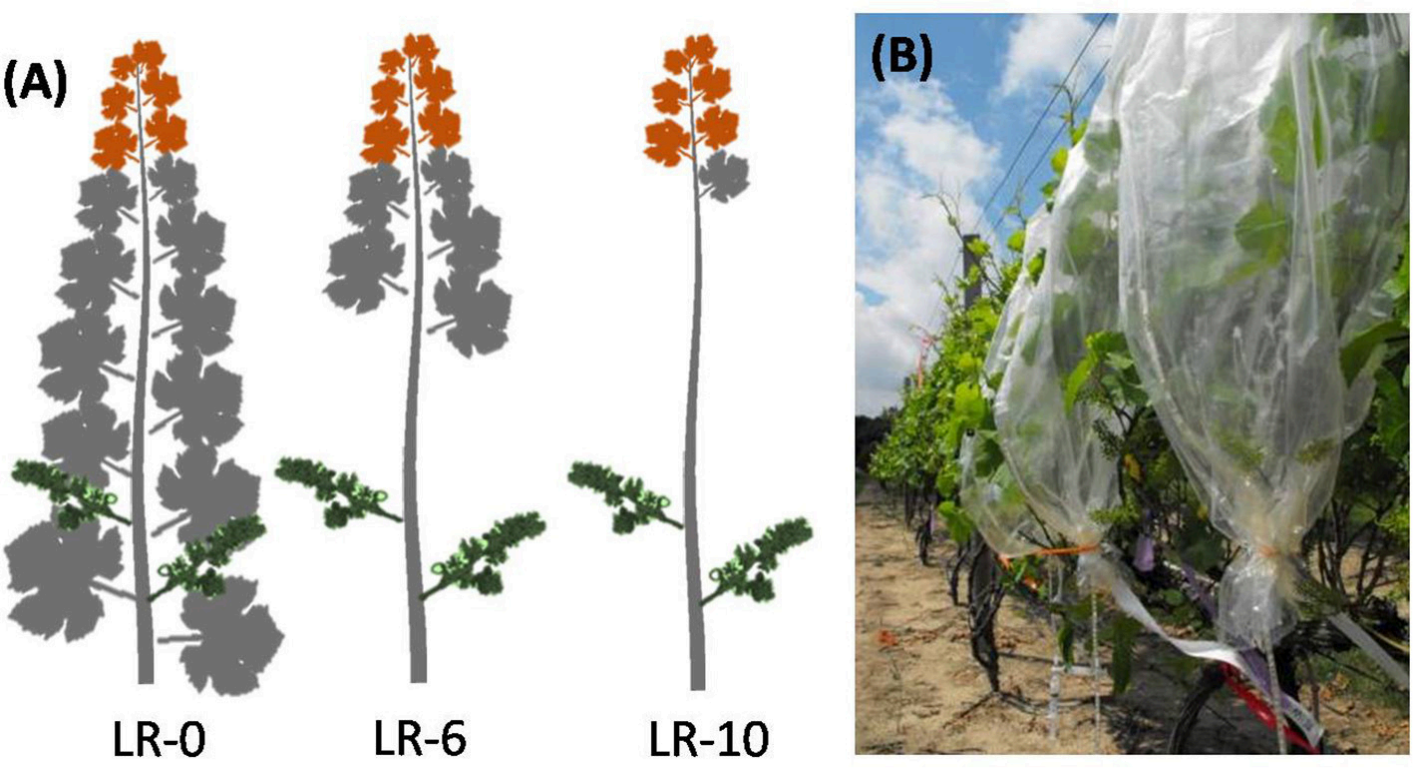
Diagrammatic representation **(A)** of early leaf removal treatments and of the different organs sampled during the pulsing study:cluster (green), fully expanded leaf (gray), shoot apex (orange). Shoots were enclosed in mylar bags **(B)** and pulsed for 30 min with ^13^CO_2_, generated by the reaction of 800 mg of Ba^13^CO_2_ (98 atom %) with 5 mL of 85% lactic acid.

The experiment was arranged in a randomized complete block design with one factor, leaf removal (LR), at three levels of defoliation (Figure 1A): no leaves removed (LR-0); main leaves removed from six basal nodes (LR-6); and main leaves removed from ten basal nodes (LR-10). Thirty-six vines were organized into four blocks and each treatment was randomly assigned to three vine per block. Additionally, a subsample of four shoots per vine was randomly chosen and tagged to make further measurements of degree of fruit-set, cluster and berry weight, and fruit chemistry. Treatments were applied at full bloom (50% of cap fall), known as developmental stage EL-23 (Lorenz et al., 1995) on June 15th. At the time of the treatment application, shoots had an average of 15 unfolded leaves.

### Estimation of fruit-set and leaf area

To better describe the fruit-set process, we defined the proportion of fruit-set as the ratio between the initial number of florets and the number of berries at harvest. Every basal bunch on each tagged shoot (*n* = 72) was photographed in the vineyard at EL-20 (onset of flowering, with 30% flower cap fallen). Twenty bunches at EL-20 were selected from guard vines and photographed in the vineyard against a dark background and then collected in poly zip bags, stored in a portable cooler and transported to the laboratory. The actual number of flowers was destructively counted. The number of florets visible in the photos was counted using Microsoft Office Paint (Windows XP; Microsoft, Redmond, WA, USA). A linear relationship (y = 2.03x, *R*^2^ = 0.86, Supplementary Figure 1) was calculated between the actual number of florets (y) and the florets counted on the photographs (x) (Supplementary Figures 2, 3).

The basal leaves removed at the time of application of LR-6 and LR-10 were transferred to the campus laboratory and leaf area (LA) was measured with a leaf area meter (LI-3050AHS, Lambda Instruments Corporation, Nebraska). Shoot length was recorded weekly, starting 2 weeks after bud-break, until July 14, the day when shoot trimming was performed. At the same time, a sample of 10 shoots, collected from guard vines, was used for estimation of the total LA per shoot. Total shoot LA was obtained via an estimating approach using the primary data of shoot length. Real measurements of shoot legth and LA was weekly assessed on guard vines. A linear correlation was built between the LA and shoot length (y = 17.51x−87.52, *R*^2^ = 0.82). The formula was used for estimation of total LA (y) of experimental vines, based on shoot length (x). The average leaf area removed with defoliation treatments LR-6 and LR-10 was subtracted from total final LA to calculate post-defoliation leaf area regrowth (Supplementary Figures 4–6).

### Yield components and fruit chemistry

Yield per vine and the total number of clusters per vine were recorded at harvest. The leaf area to yield ratio was calculated multiplying the single shoot LA measured at trimming (see previous paragraph) with the number of shoots of the same vine, obtaining the LA per vine. The LA per vine was divided with the yield per vine to calculate the LA to yield ratio (m^2^/kg). Basal clusters from selected and tagged shoots were weighed, then berries were separated from the rachis and their total number and weight were recorded, then the berries were returned to the sample poly bag and saved for subsequent chemical assessment. Basic fruit chemistry and color were determined as described by Iland et al. (2004) mixing all the components of the berries (skin, pulp, and seeds). Number of seeds per berries were not recorded and no chemical analyses were performed on the seeds. We extracted ~20 mL of juice from each bunch sample for analysis of both must total soluble solids (TSS) using an Atago PAL-1 Refractometer (Atago USA, Inc.) and pH (Thermo Scientific Orion 370 pH meter; Beverly, MA, USA). For determination of titratable acidity (TA), 10 mL of juice was titrated against a standardized 0.1N NaOH solution to a pH of 8.2 in an automated titrator coupled to an auto-sampler and control unit (Titroline 96; Schott-Geräte, Mainz, Germany) and expressed as g/L of tartaric acid equivalents. Anthocyanins and phenolic substances were measured by the total phenol assay, using UV–VIS (Iland et al., 2004). One hundred berries stored at −30°C were partially thawed prior to grinding in a tissue homogenizer (Model PT 10/35; Brinkmann Instruments, Luzern, Switzerland) at a speed of four on the manufacturer's scale for about 1 min. Samples were ground while maintained in an ice bath to minimize oxidation, and the concentration of anthocyanins per gram of berry mass and the absorbance units of phenolic substances per gram of fresh berry were measured with a spectrophotometer (UV-1800; Shimadzu, Kyoto, Japan) (Iland et al., 2004).

### Gas exchange measurements

Leaf assimilation (P_n_), and stomatal conductance (g_s_) were measured with a portable open system gas analyzer (CIRAS-2, PPS Co. Ltd., England). Twelve vines per treatment were chosen from the eastern side of the cordon. Measurements were taken on leaves located on the 11th node from the base, common position in all the treatments, between 1000 and 1300 h, 7 days after defoliation (21 June 2011). The system was equipped with a 6.25 cm^2^ leaf chamber and all readings were taken at ambient RH with an air flow adjusted to 350 mL/min. Measurements were taken under saturating light conditions (PAR> 1400), with a CO_2_ reference point set at 380 ppm. Then, the entire shoot photosynthesis was estimated, considering the leaf area of the different treatments at the moment of the gas exchanges measurement (leaf P_n_ x shoot LA).

Diurnal trends of gas exchanges were recorded on 12 replicates per treatment. Two shoots per vine were selected from the eastern side of the cordon and measurements of P_n_, g_s_, were taken on leaves located on the 11th node from the base at 1000, 1200, 1400, 1600, and 1800 h on 6 July 2011. Then, for each replicate, the daily integral of photosynthesis, expressed as mmol CO_2_/m^2^, was calculated after Hendrickson et al. (2004) using the macro option “area below curves” available in SigmaPlot 11 (Systat Software Inc.).

### Application of ^13^C

Four vine replicates per treatment were used for quantification of carbon translocation along main shoots 1 week after defoliation on 22 June 2011. Three shoots per vine were randomly selected and individually enclosed in mylar bags (Figure 1B). Each shoot was pulsed for 30 min with ^13^CO_2_, which was generated by the reaction of 800 mg of Ba^13^CO_2_ (98 atom %) with 5 mL of 85% lactic acid. Pulsing was done between 1000 and 1400 h under clear sky conditions. After 30 min of pulsing, the mylar bags were removed and shoots were exposed to the ambient air. Twelve samples per treatment of shoot apex plus immature leaves and fully expanded leaves (~3 cm^2^ of tissue per sample), and the entire clusters were collected 1 h after ^13^CO_2_ labeling from the first shoot. Twenty-four hours later, one shoot per vine was completely harvested, while the second and third shoots were harvested 3 and 7 days after ^13^CO_2_ labeling, respectively. Additionally, three shoots from non-labeled vines were collected for ^13^C natural abundance determination. Harvested shoots were divided into shoot apex with immature leaves, fully expanded leaves and clusters sub-samples. The parts of the shoots were oven-dried at 70°C for 2 days and their dry weights recorded. Dry tissues were ground to a fine powder with mortar and pestle and sieved with mesh size 40. Approximately 1.5 mg of each sample was encased in small tin capsules, placed in trays, and sent to the Stable Isotope Facility, UC Davis, California for ^13^C-analysis. The ^13^C atom excess % and the percentage of ^13^C distribution per organ were calculated as described by Morinaga et al. (2003). For each replicate, ^13^C partitioning during the pulsing study was evaluated also as an hourly difference between pulsed ^13^C (P–^13^C) and natural abundance of ^13^C (N–^13^C). An “apex sink strength” coefficient at the time of fruit-set was calculated as the average of the percentages of ^13^C allocated in the apices between 24 and 168 h during the time course pulsing study. The calculated apex sink strength so calculated was used to build linear regressions with fruit-set and shoot photosynthesis, using SigmaPlot 11 (Systat Software Inc.).

### Statistical analysis

Data were analyzed using one-way ANOVA in PROC MIXED procedure, SAS 9.3 (SAS Institute, Cary, NC, USA). When the treatment effect was statistically significant at α = 0.05, all-pairwise comparisons among the treatments were conducted using Tukey's HSD. Regression analysis was performed using SigmaPlot 11 (Systat Software Inc.). Photo-assimilates partitioning and diurnal measurements of P_n_ and g_s_ were analyzed using the REPEATED statement function in PROC MIXED. When the treatment effect was found to be statistically significant at α = 0.05, all-pairwise comparisons among the treatments were conducted using the *t*-test.

## Results

### The effect of defoliation on canopy growth and vine balance

**Figure 2:**
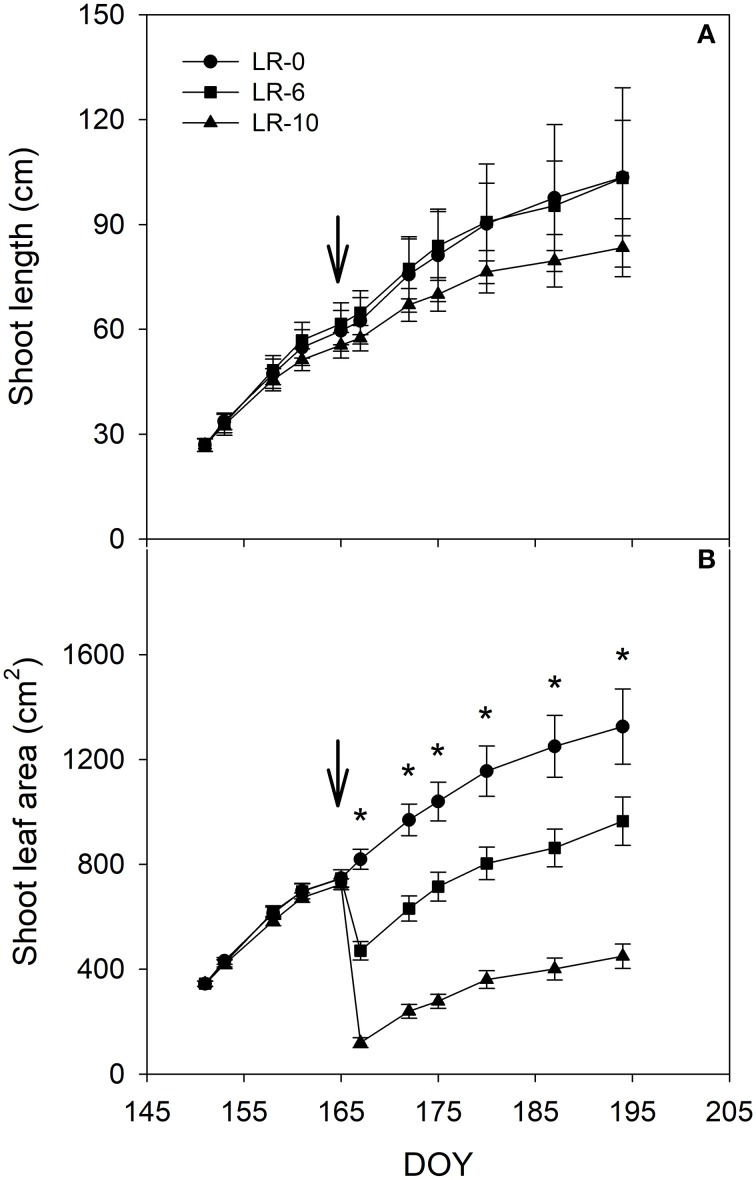
Shoot length **(A)** and shoot leaf area (main leaves, **B**) in relation to different intensity of leaf removal. Each point is the mean of 48 values, vertical bars represent standard errors. The arrow represent grapevine full bloom phenological stage and the time of the leaf removal treatment. Leaf area was measured until the day before canopy trimming on July 14. Symbol (^*^) represent significant difference at *P* < 0.05. LR-0, no leaves removed; LR-6, leaves removed from 6 basal nodes; LR-10, leaves removed from 10 basal nodes at bloom; DOY, Day Of Year.

Defoliation severely impacted shoot leaf area that was reduced 47 and 86% in LR-6 and LR-10, respectively, as compared to pre-treatment levels (Table 1). The defoliation treatments also affected shoot growth and LA development during the growing season (Figure 2). LR-10 caused a deceleration of shoot elongation (Figure 2A) and pre-trimming main shoot length was 84 vs. 101 cm in LR-0 and LR-6 vines, however differences were not significant. At the time of shoot trimming, the main leaf area in LR-6 and LR-10 was 73 and 34%, compared to the control (LR-0), respectively. Vine balance, indexed as LA to yield ratio, was impacted by leaf removal in both LR-6 and LR-10 (Table 1). LR-6 had a leaf area to yield ratio significantly lower than LR-0 (−0.27 m^2^/kg). Similarly, LR-10 vines showed a significant reduction of the ratio (−0.21 m^2^/kg than LR-0), whereas no difference was found if compared to LR-6.

**Table 1:** Impact of leaf removal on shoot leaf area and vine balance.

**Treatment^a^**	**Shoot leaf area before treatments application^b^ (cm^2^)**	**After treatments application**^b^			**Shoot leaf area before canopy trimming^b^ (cm^2^)**	**Leaf area to yield ratio (m^2^/kg)**
		**Removed leaf area (cm^2^)**	**Removed leaf area (%)**	**Retained leaf area (cm^2^)**		
LR-0	747 a	0 c	0 c	747 a	1326 a	0.87 a
LR-6	766 a	359 b	47 b	407 b	965 b	0.60 b
LR-10	724 a	619 a	86 a	105 c	449 c	0.66 b

*Means within the column followed by the same letter are not significantly different at P < 0.05 by Tukey's HSD test*.

a *LR-0, no leaves removed; LR-6, leaves removed from 6 basal nodes; LR-10, leaves removed from 10 basal nodes at bloom*.

b *Treatments were applied on 15 June (DOY 166), coinciding with full bloom. Trimming was executed on 14 July (DOY 195)*.

### Impact on fruit-set, yield components, and fruit chemistry

**Figure 3:**
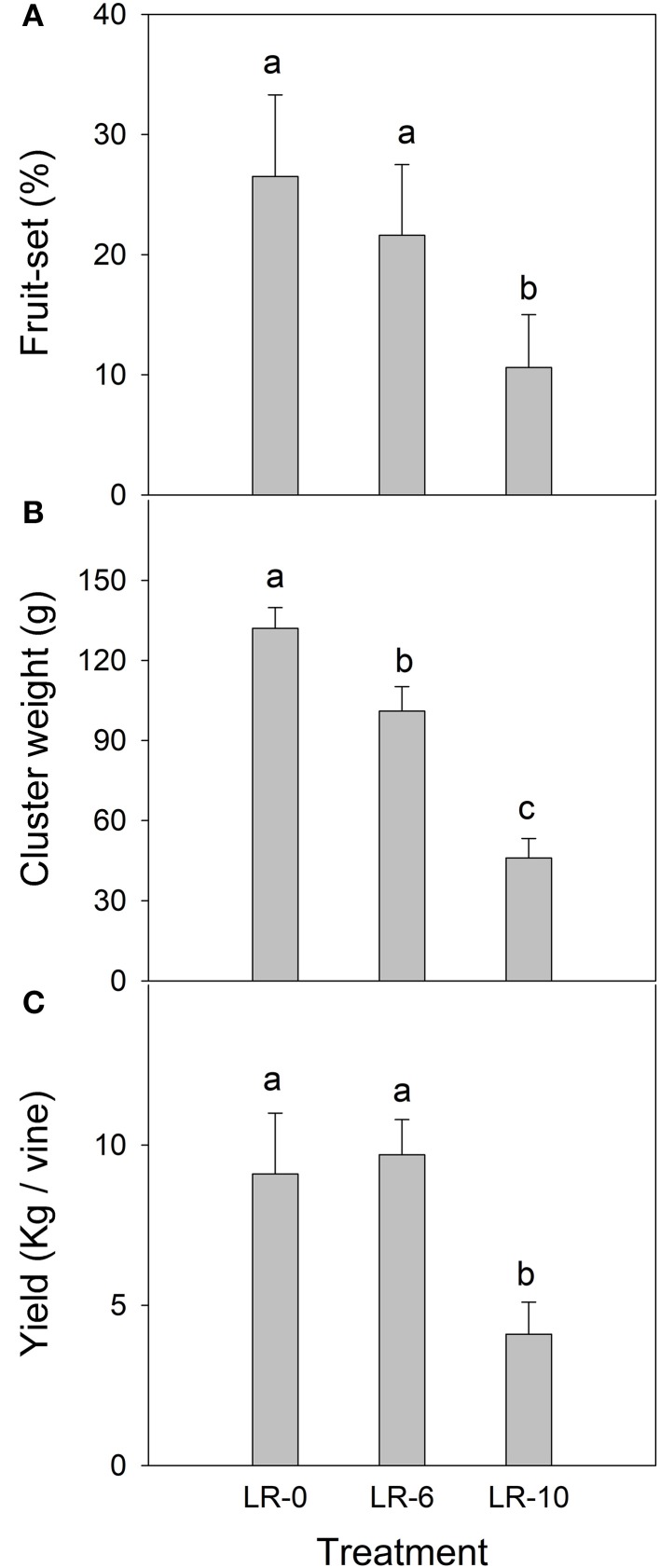
Fruit-set **(A)**, cluster weight **(B)**, and yield **(C)** of vines subjected to different intensity of early leaf removal. Each point is the mean of 12 values. Different letters indicate significant effects of the treatment at *P* < 0.05 (Tukey's HSD test). LR-0, no leaves removed; LR-6, leaves removed from 6 basal nodes; LR-10, leaves removed from 10 basal nodes at bloom. Vertical bars represent standard deviation (*n* = 24).

While number of florets per cluster was uniform among treatments before defoliation (Table 2), LR-10 reduced fruit-set at EL-38 (harvest) by 60% (Figure 3A). Consequently, LR-10 significantly impacted number of berries per cluster (−24%, Table 2) and cluster weight (−65%, Figure 3B). In LR-6, the treatment did not affect fruit-set (Figure 3A) and number of berries per cluster (Table 2); despite the values being slightly lower, they were not significantly different from LR-0. However, cluster weight was significantly reduced (−23%) by LR-6 (Figure 3B). Berry weight was unaffected by any of the treatments (Table 2). Early leaf removal caused a reduction in yield per vine in treatment LR-10 (4.1 kg vs. ≅ 9 kg scored by C and EL-6 vines), whereas no difference was observed in LR-6, when compared to LR-0 (Figure 3C).

**Table 2:** Impact of leaf removal on cluster components, basic fruit chemistry, and phenolic content.

**Treatment^a^**	**Number of florets per cluster**	**Number of berries per cluster at EL-38**	**Berry weight (g)**	**TSS (Brix)**	**pH**	**Titratable acidity (g/L)**	**Anthocyanin (mg/g)**	**Phenolics (a.u./g)^b^**
LR-0	425 a	113 a	1.08 a	20.9 b	3.46 b	6.09 a	0.34 a	0.95 b
LR-6	442 a	106 a	1.04 a	21.9 b	3.49 b	5.49 ab	0.29 a	0.86 b
LR-10	443 a	73 b	0.98 a	24.0 a	3.69 a	4.95 b	0.37 a	1.20 a

Means within the column followed by the same letter are not significantly different at P < 0.05 by Tukey's HSD test.

a *LR-0, no leaves removed; LR-6, leaves removed from 6 basal nodes; LR-10, leaves removed from 10 basal nodes at bloom*.

b *a.u. = absorbance units*.

The highest must soluble solids and pH were found in LR-10 (Table 2). This treatment also showed significantly lower titratable acidity (TA) when compared to non-defoliated vines (Table 2). LR-6 did not affect basic fruit chemistry in any of the parameters evaluated. No difference between treatments was found in the concentration of anthocyanins. However, LR-10 significantly increased phenolics as compared to LR-0 and LR-6 (Table 2).

### The effect of defoliation on gas-exchanges and carbon distribution

LR-10 had higher P_n_ when compared to the other treatments (Table 3) and was significantly increased by 42% when compared to LR-0. Similarly, LR-6 increased P_n_ by 12%, however this was not statistically different from the control. Both defoliation severities scored higher g_s_ than non-defoliated vines. An opposite outcome was found when P_n_ was expressed on a shoot leaf area basis. In fact, LR-10 evinced a significant decrease on P_n_/shoot vs. LR-0 (−65%), whereas the P_n_/shoot registered in LR-6 was 27% lower, when compared to the non-defoliated control.

**Table 3:** Impact of early leaf removal on leaf assimilation (P_n_) and stomatal conductance (g_s_), at 6 days after full bloom and leaf removal.

**Treatment^a^**	**P_n_ (μmol CO_2_ m^−2^ s^−1^)**	**g_s_ (mmol m^−2^ s^−1^)**	**P_n_/shoot (μmol CO_2_ s^−1^)**
LR-0	9.7 b	241.7 b	0.94 a
LR-6	10.9 b	284.2 a	0.69 b
LR-10	13.8 a	301.6 a	0.33 c

Means values were based on 8 replicates. Means within the column followed by the same letter are not significantly different at P < 0.05 by Tukey's HSD test.

a *LR-0, no leaves removed; LR-6, leaves removed from 6 basal nodes; LR-10, leaves removed from 10 basal nodes at bloom*.

**Figure 4:**
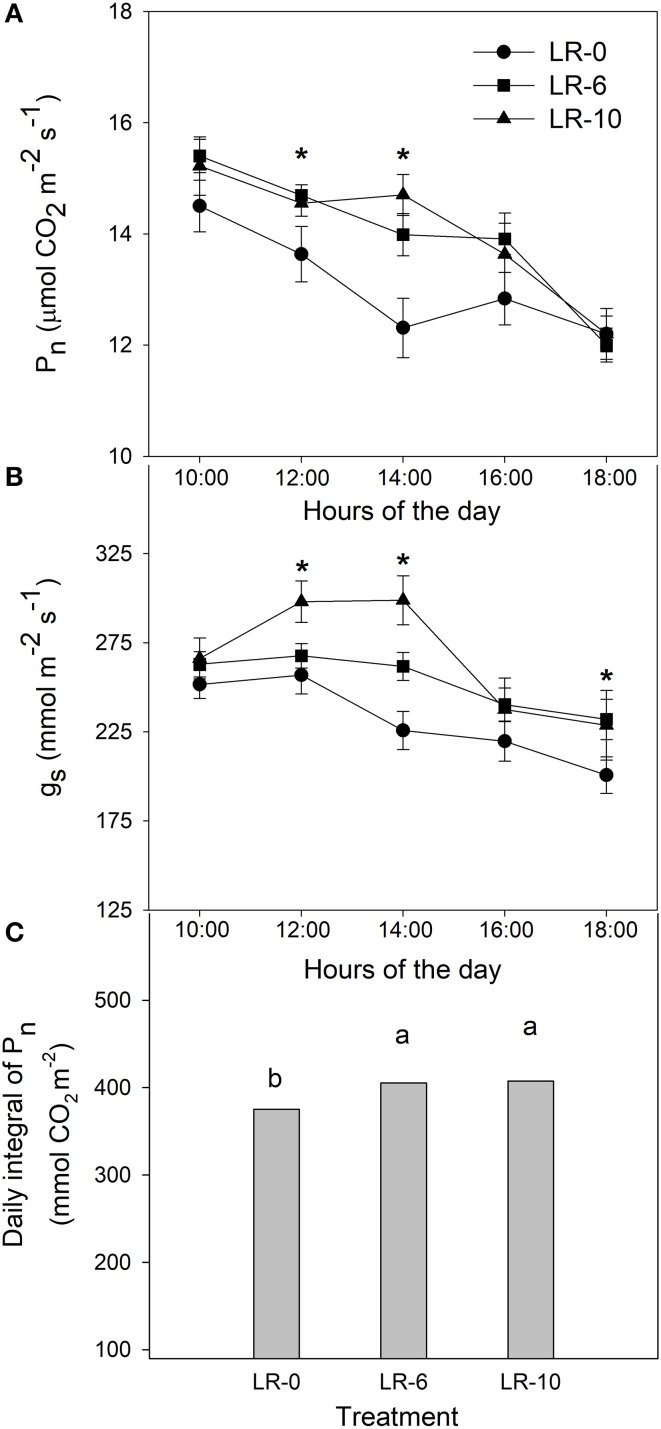
Effects of early leaf removal on daily leaf net CO_2_ assimilation rate **(A)**, stomatal conductance **(B)** and daily integral of P_n_ three weeks after full bloom **(C)**. Means were based on 3 replicates. Error bars represent standard error of the mean (SE). Symbol (^*^) represent significant difference at *P* < 0.05. Different letters indicate significant effects of the treatment at *P* < 0.05 by Tukey's HSD test. LR-0, no leaves removed; LR-6, leaves removed from 6 basal nodes; LR-10, leaves removed from 10 basal nodes at bloom.

P_n_ and g_s_ were also measured as diurnal trends (Figure 4). At 1200 and 1400 h, both LR treatments led to a much higher P_n_ than LR-0 (+1.06 μmolCO_2_/m^2^/s and +2.03 μmolCO_2_/m^2^/s at 1200 and 1400 h, respectively). Stomatal conductance in LR-0 was generally lower than any defoliation treatment, while LR-10 showed higher g_s_ than LR-6 for readings taken at 1200 and 1400 h (Figure 4). When considering the daily integral of photosynthetic activity, LR-10 had a significantly higher assimilation rate than undefoliated vines (+ 32 mmol/CO_2_/m^2^). LR-6 showed rates similar to the ones observed in LR-10.

**Figure 5:**
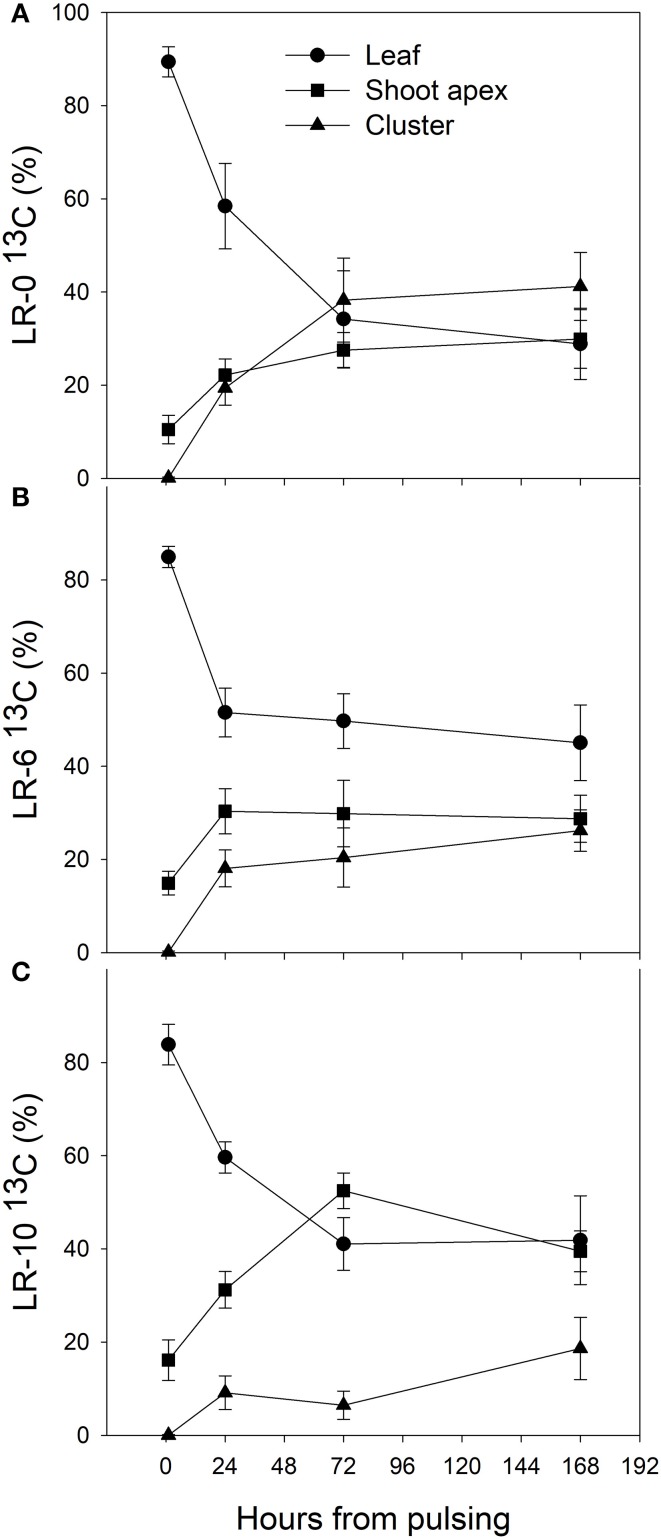
Distribution (%) of ^13^C in leaf, shoot apex and cluster of grapevine subjected to different intensity of leaf removal from 1 to 168 h after ^13^CO_2_ pulsing. Each point represents the mean of 4 values ± SE. Hour 0 represents the moment of pulsing and correspond to 7 days after leaf removal and therefore 7 days after full bloom. Symbol (^*^) represent significant difference at *P* < 0.05. LR-0, no leaves removed **(A)**; LR-6, leaves removed from 6 basal nodes **(B)**; LR-10, leaves removed from 10 basal nodes at bloom **(C)**.

**Figure 6:**
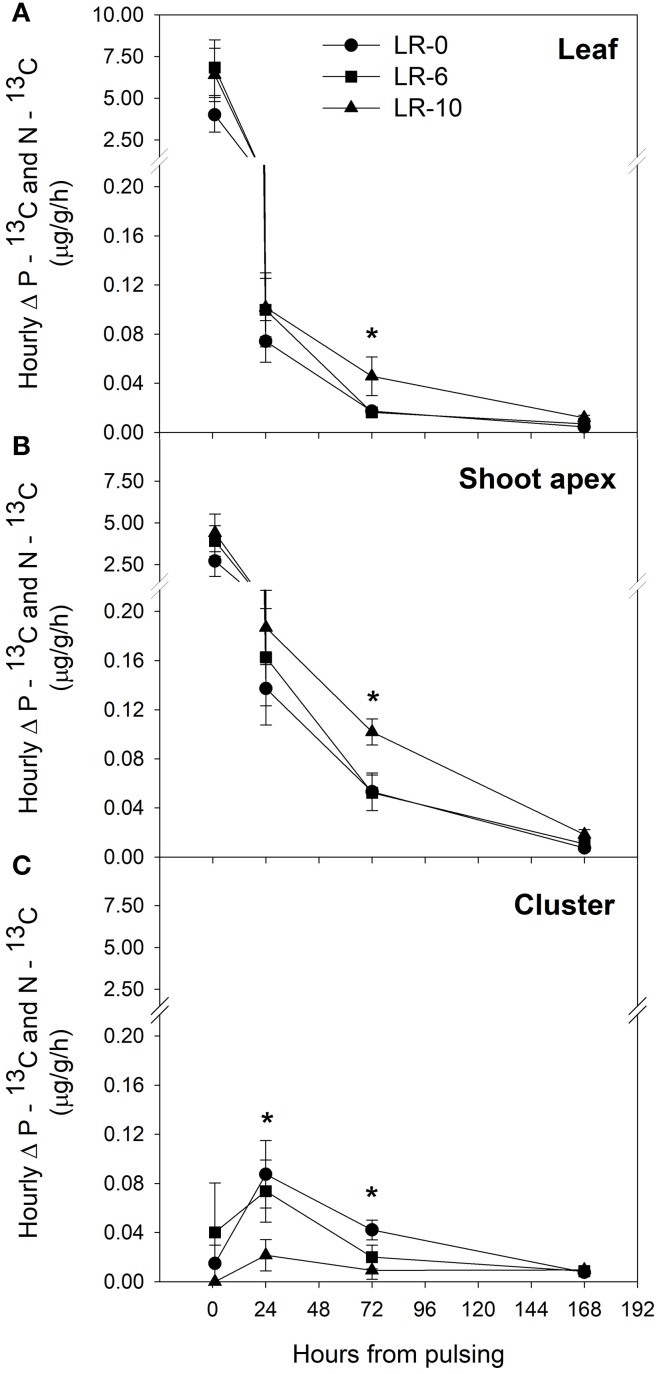
Hourly difference (Δ) between the pulsed ^13^C (P–^13^C) and ^13^C natural abundance (N–^13^C) in leaf **(A)**, shoot apex **(B)**, and cluster **(C)** after 1 to 168 h from ^13^CO_2_ pulsing in grapevines subjected to different leaf removal treatments. Each point represents the mean of 4 replicates ± SE. Zero hours from pulsing represents the moment of pulsing and correspond to 7 days after leaf removal and therefore 7 days after full bloom. Symbol (^*^) represent significant difference at *P* < 0.05.

**Figure 7:**
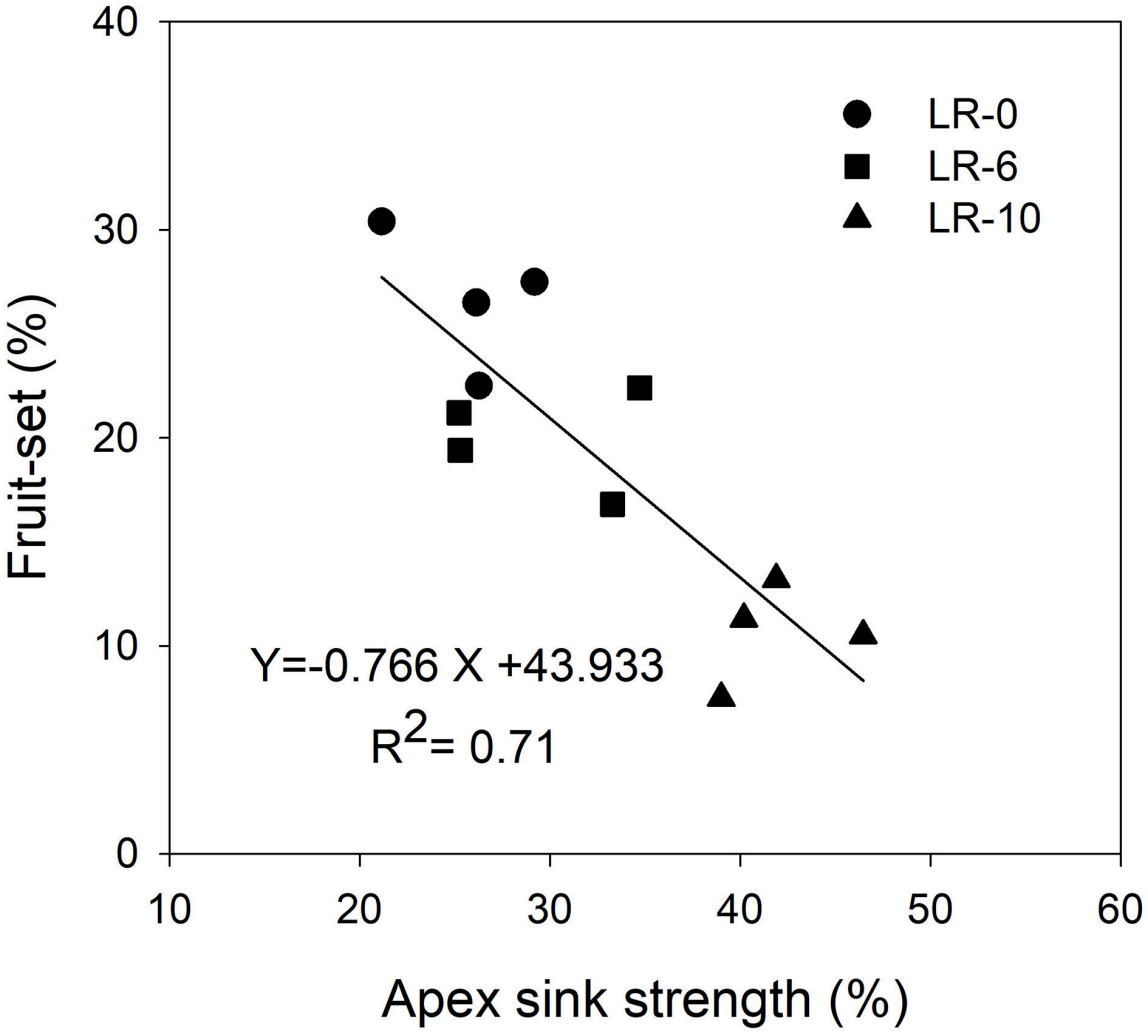
Linear regression between shoot apex sink strength and cluster fruit-set at harvest. Shoot apex sink strength was calculated as the mean of ^13^C percentage allocation during the pulsing study (from 7 to 14 days after bloom).

**Figure 8:**
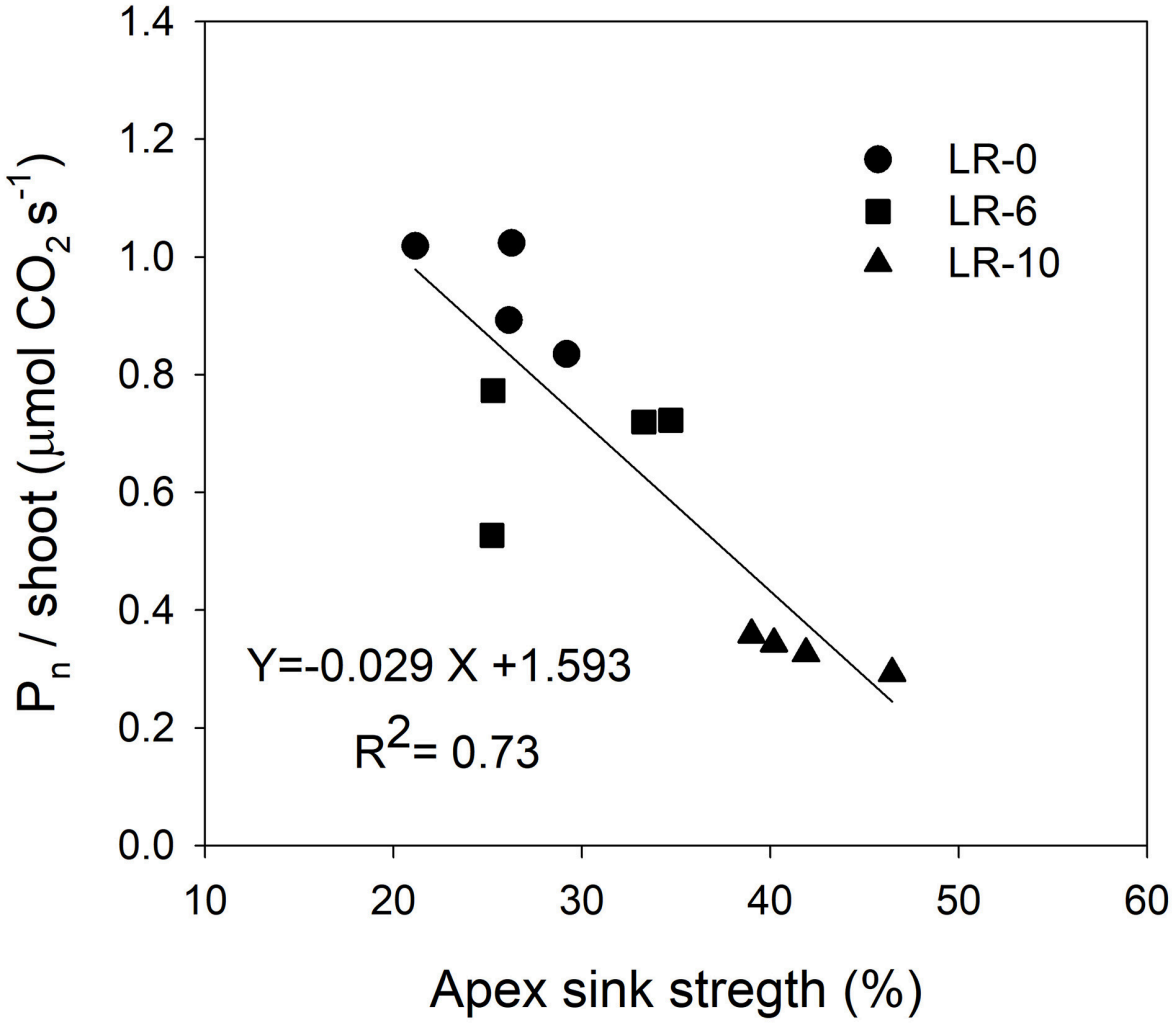
Linear regression between shoot apex sink strength and shoot net photosynthesis (P_n_/shoot). Shoot apex sink strength was calculated as the mean of ^13^C percentage allocation during the pulsing study (7 to 14 days after bloom).

At the beginning of the study, the majority of carbon was allocated to the main leaves in all treatments (Figure 5). In LR-0, a week after pulsing, the three organs had a fairly even partitioning of ^13^C (Figure 5A). At the same time, in LR-6, main leaves had a distinctly higher ^13^C % (about 50%) than shoot apex and clusters (Figure 5B). Conversely, LR-10 showed, at 7 days after pulsing, minimal ^13^C % distribution into clusters whereas allocation to main leaves and apices set at about 40%. At 7 days after pulsing, ^13^C recover in the different organs was quite similar to that measured at the intermediate date, with the exception of ^13^C % allocated to clusters in the LR-10 treatment that significantly increase. During the pulsing study, LR-10 allocated significantly less ^13^C % in the clusters when compared to other treatments (9 vs. 19% allocated by LR-0 at 24 h from the pulsing, 6 vs. 38% at 72 h), whereas a significantly higher ^13^C % was allocated to shoot apices at 72 h from the pulsing (52 vs. only 30% allocated by LR-0), that became the most important sink for ^13^C. Similarly, when considering the ^13^C accumulation rate (Figure 6), LR-10 promoted higher rates on leaf and shoot apices and lower rates on clusters, when compared to LR-0. In particular, between 24 and 72 h from the pulsing, LR-10 maintained higher rates on leaves and apices than the other treatments (+0.05 μg/g/h, when compared to LR-0). Conversely, between 1 and 72 h from the pulsing accumulation rates in clusters were much lower in LR-10 than any other treatment. Fruit-set was negatively correlated with the apex sink strength (Figure 7). A linear regression described the correlation between the two parameters (*R*^2^ = 0.71). Similarly, apex sink strength resulted in a negative correlation (*R*^2^ = 0.73) with the shoot photosynthesis (Figure 8).

## Discussion

Fruit set, defined as the ratio between set berries and initial flower number, is a primary determinant of final yield and it takes place in a specific moment of the annual growing cycle, when several strong active sinks coexist on the vine and carbon reserves are at their seasonal minimum level during the season (Coombe, 1959; Candolfi-Vasconcelos et al., 1994; Palliotti et al., 2011). Green inflorescences are photosynthetically active, but they are far from being capable of supporting their own development, especially because their chlorophyll content substantially decreases after full bloom (Palliotti and Cartechini, 2001; Lebon et al., 2005). Therefore, the growth of set berries primarily relies on carbon assimilated by source leaves (i.e., those having reached at least 30–40% of their final size according to Poni et al., 1994), located at the adjacent nodes right below or above the cluster (Hale and Weaver, 1962; Motomura, 1990). Inadequate supply of carbon to the inflorescence and developing cluster, often results in poor fruit-set, due to abortion of embryos in set berries followed by berry drop (Coombe, 1959; Candolfi-Vasconcelos and Koblet, 1990). Several studies reported that source limitations at bloom interfere with fruit-set and fruit formation, in grapevines like in other annual or perennial species (Poni et al., 2009; Galiano et al., 2011; Palliotti et al., 2011; Vargas-Ortiz et al., 2013, 2015). Even if the role of carbon limitation is well-known, the shoot strategy in prioritizing carbon allocation to different sinks under carbon starvation conditions induced by defoliation, is not clearly established. Considering the recently published research on leaf removal applied at bloom, the significant lower percentage of fruit-set found in LR-10 is not surprising (Figure 3), whereas it remains to be explained why the LR-6 treatment, that still removed 47% of the pending leaf area (Table 1), had no significant effects on either fruit-set and final yield.

The defoliation-induced compensation in terms of gas exchange is likely due to a reduction in feedback inhibition after leaf removal, resulting in P_n_ and g_s_ increase in both defoliation treatment (Figure 4). These results are consistent with previous experiments on defoliation at fruit set (Hunter and Visser, 1988; Candolfi-Vasconcelos and Koblet, 1991). Despite this, the increase in P_n_ was not sufficient to fully compensate the loss of shoot carbon assimilation due to leaf removal. In fact, shoot carbon assimilation was severely curtailed in LR treatments in accordance with previous experimentation studying LR on whole canopy photosynthesis (Poni et al., 2008). The drastic reduction of leaf area in LR-6 and LR-10 and subsequently of P_n_/shoot occur in a key-moment of energy demand for fruit-set (Lebon et al., 2005). Therefore, in LR-10 the berry set took place in a condition of relevant carbon starvation and this led to the reduced fruit–set, cluster weight and yield reported. These results are consistent with previous researches carried out in the same environment, where over a 2-years study defoliation of six to eight basal nodes at full flowering was deemed a leaf removal threshold able to regulate fruit-set and consequently cluster compactness in Pinot noir grapevines under cool climate conditions (Acimovic et al., 2016). The reduction of shoot carbon assimilation induced by LR treatments was coupled with a consistent change in carbon partitioning: ^13^C percentage allocation was remarkably lower in clusters of LR-10 72 h after the pulsing but ^13^C allocation to the shoot apex was significantly higher according with LR intensity (Figure 5). This indicates an increase of the relative sink strength of shoot apex in LR-10 (Figure 5). Notably, in LR-10 the shoot apex becomes the most important sink within the shoot for ^13^C at 72 h after pulsing, whereas in LR-0 and LR-6 it represents a secondary destination for assimilates all along the fruit-set process. The increase of relative carbon translocation to shoot apex can be partly explained by the distance from source leaves that plays an important role in the allocation of new synthetized assimilates toward the sink organs of the shoot (Quinlan and Weaver, 1970; Motomura, 1990).

Grapevine flowers are weak competitors for carbon, especially in source-limiting conditions induced by leaf removal (Keller, 2010). However, assimilate transport is a dynamic process, with a higher degree of adaptation in relation to vine phenological stages and the different needs of the sinks, imposed by the cultural practices and the environment. This results in a hierarchy of relative priorities between the shoot sinks and sources, and this hierarchy is dynamic and sensitive to vine manipulations and climatic conditions (Keller, 2010). Fruit set was negatively correlated to the shoot apex sink strength (Figure 7). Our results suggest that the shoot apex competition for carbon, plays a pivotal role in reducing the amount of carbon allocated to reproductive activity: relative strength of shoot apex sink was negatively correlated with the global assimilation of the shoot (Figure 8). In this case, carbon allocation to vegetative organs, is favored at the detriment of the reproductive activity. This is confirmed by the fact that LR-6 treatment had a modest effect on fruit set but no effect on shoot growth, while LR10 significantly reduced fruit set and slightly reduced shoot vegetative growth. Overall, our experiment suggests a dynamic effect of the removal of carbon source via prioritization of active sinks, indicating that the fruit set decrease, is not only function of the overall limitation in the amount of carbon assimilated by shoot, as hypothesized in literature (Poni et al., 2008; Palliotti et al., 2011).

## Conclusion

Carbon assimilation depression, caused by the removal of a large percent of the assimilatory organs at bloom, had a major impact on carbon allocation to sinks and reproductive activity. Reduction of carbon assimilation per shoot was linearly correlated with the priority that shoot apex gained in term of sink destination, which, in turn, was negatively correlated with fruit set. This process can therefore be associated with the relative sink strength of developing vegetation, in addition to the previously understood total decrease of carbon assimilation. This information aids the understanding of relationship between source availability, carbon allocation, and fruit set, and it can be useful to determine fruit set inconsistencies as a result of photoassimilation, environmental factors, and carbohydrates reserve status.

## Author contributions

PSi and DA planned and performed the experiment. DA and TF elaborated data, ran statistical analysis, and arranged tables and figures. TF, PSa and ST wrote the first draft of the manuscript. AP and SP critically revised the manuscript.

### Conflict of interest statement

The authors declare that the research was conducted in the absence of any commercial or financial relationships that could be construed as a potential conflict of interest.

## Supplementary material

Supplementary Figure 1 Linear regression between the number of flowers counted on clusters sampled from guard vines and the number of flowers counted on pictures of the same clusters, shot in field conditions right before their sampling. Pictures shooting and clusters sampling was performed at full bloom (15th June), before application of defoliation on experimental vines.

Supplementary Figures 2, 3 Correlation analysis between the actual number of florets (y) and the florets counted on the photographs (x); the equation is reported in the text.

Supplementary Figures 4–6 Relationship between the LA per shoot (y) and shoot length (x) used for estimation of total LA.
