# Supplementary figures and images for: Changes in Within-Shoot Carbon Partitioning in Pinot Noir Grapevines Subjected to Early Basal Leaf Removal
Source: Front Plant Sci. 2018 Aug 3;9:1122. doi: 10.3389/fpls.2018.01122 (PMC6085605; doi:10.3389/fpls.2018.01122)

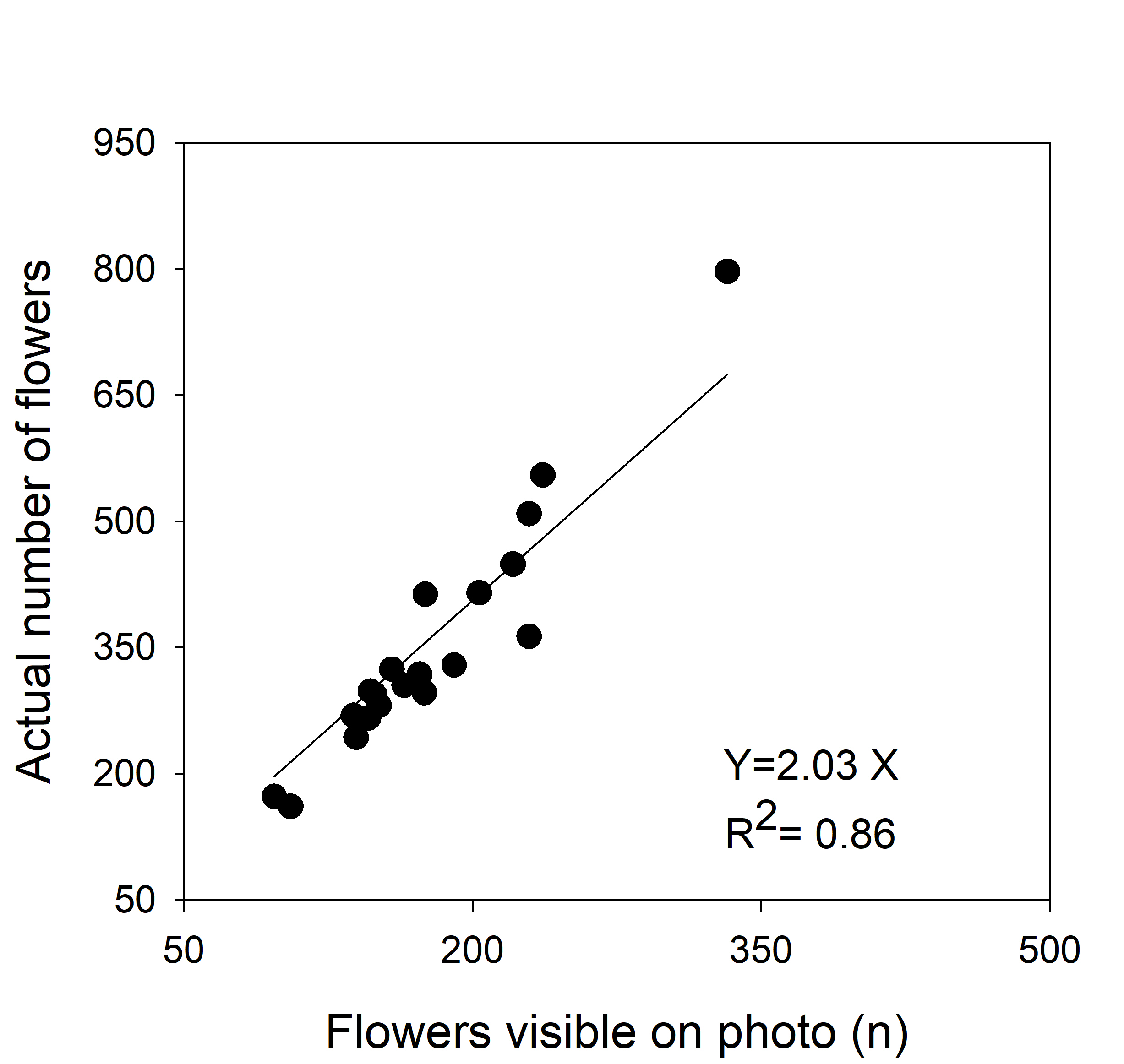

Supplement: Supplementary Figure 1 — Linear regression between the number of flowers counted on clusters sampled from guard vines and the number of flowers counted on pictures of the same clusters, shot in field conditions right before their sampling. Pictures shooting and clusters sampling was performed at full bloom (15th June), before application of defoliation on experimental vines. [file Image_1.JPEG]

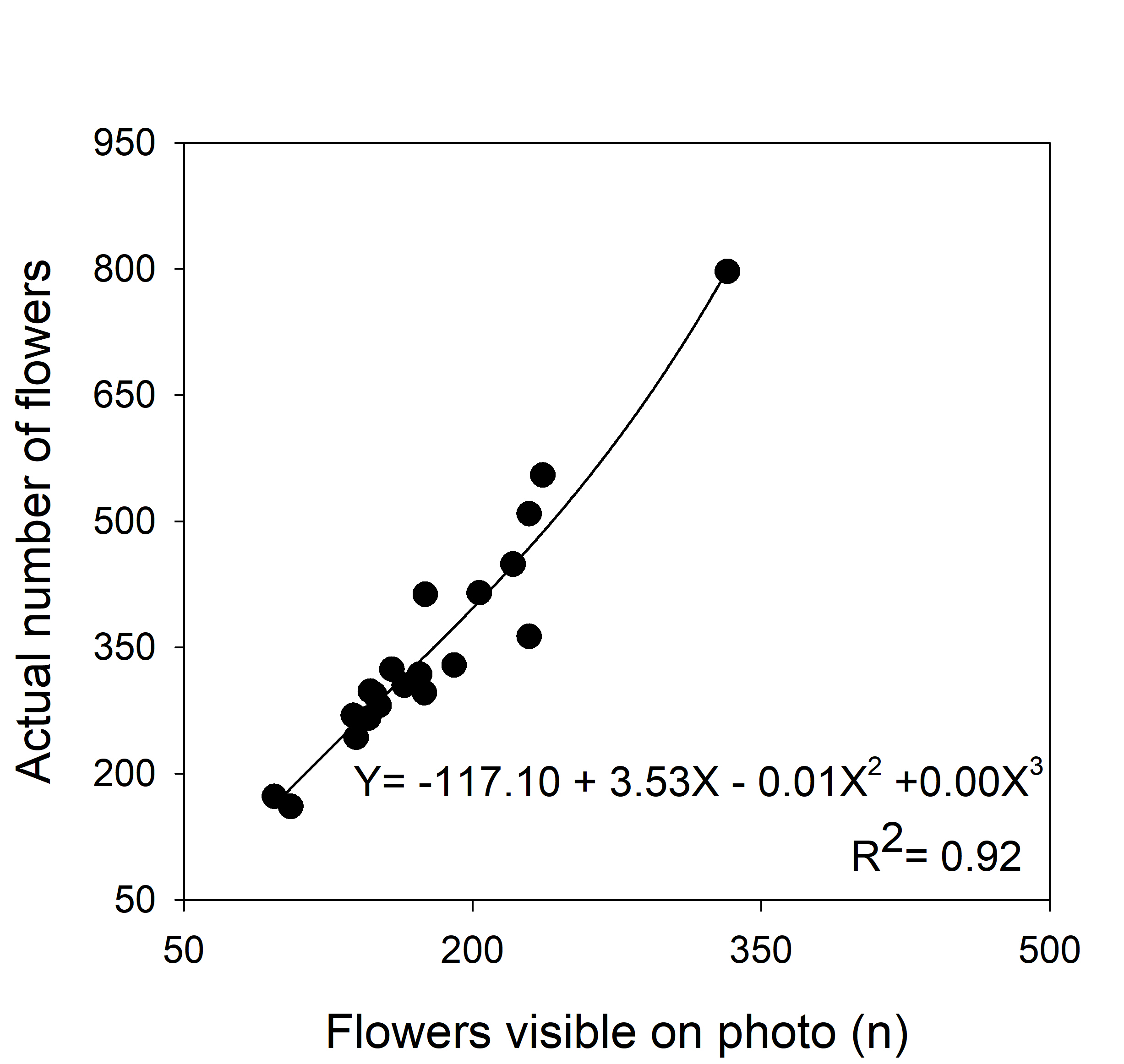

Supplement: Supplementary Figures 2, 3 — Correlation analysis between the actual number of florets (y) and the florets counted on the photographs (x); the equation is reported in the text. [file Image_2.JPEG]

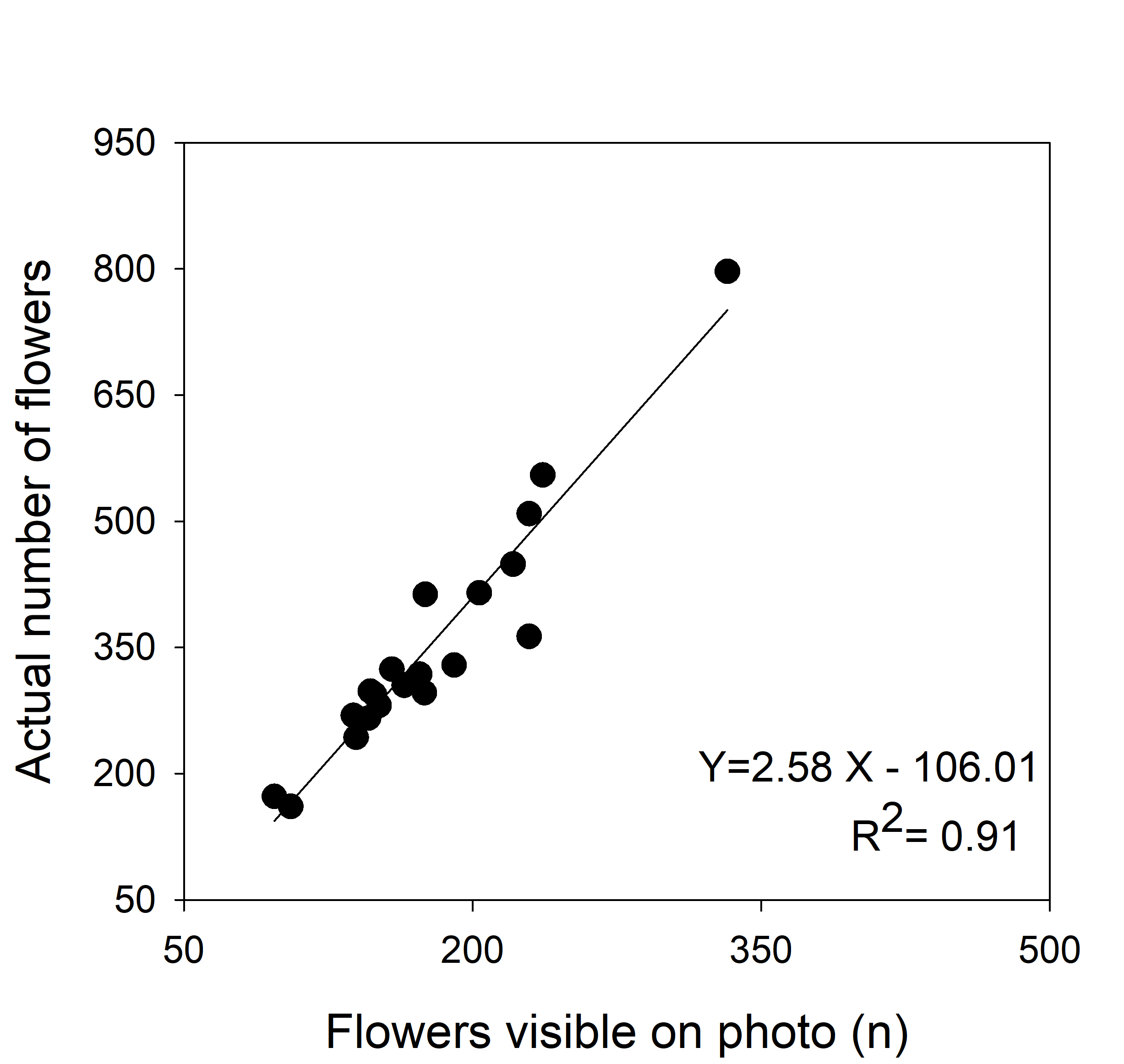

Supplement: Supplementary file 3 [file Image_3.JPEG]

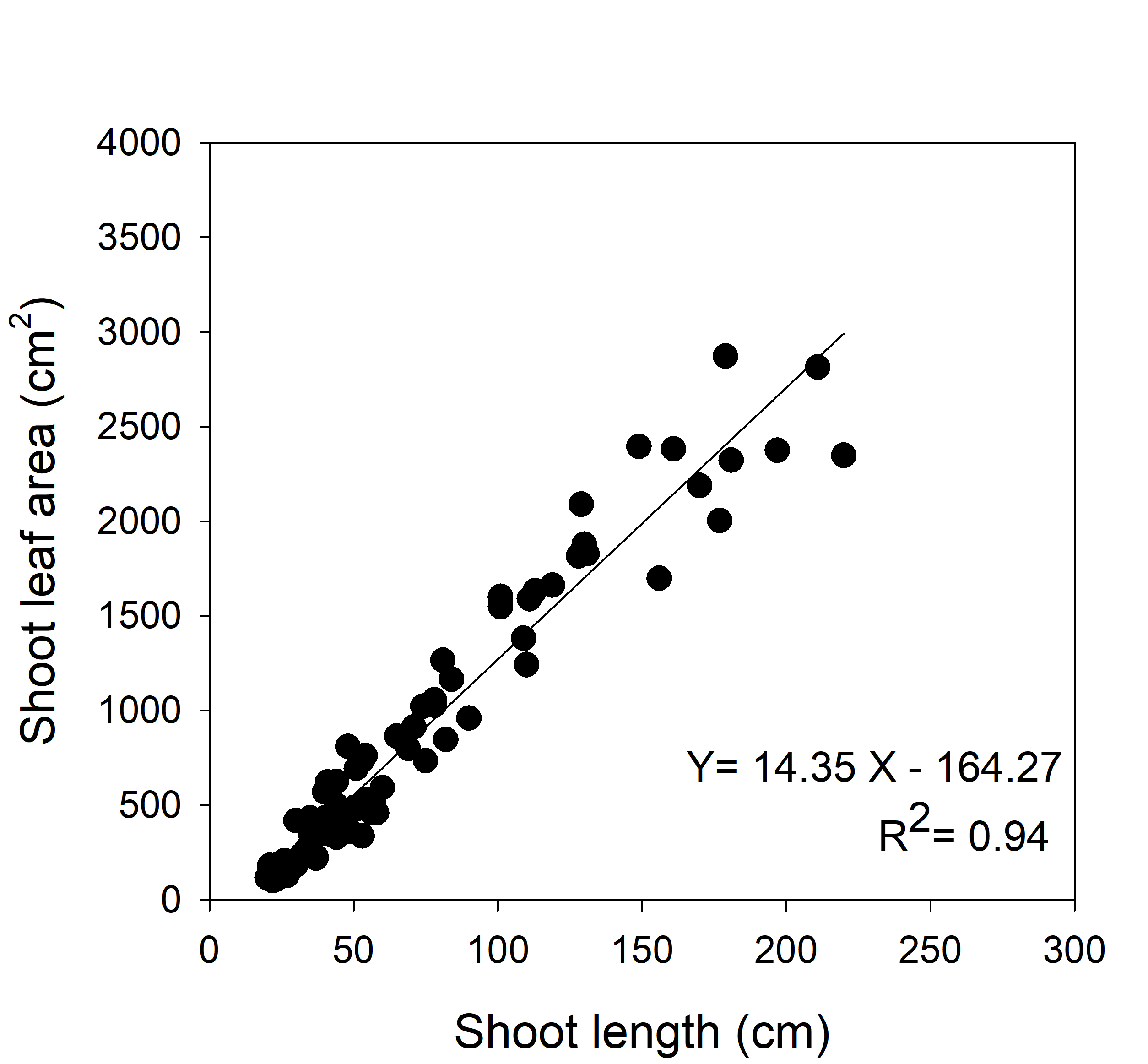

Supplement: Supplementary file 5 [file Image_5.JPEG]

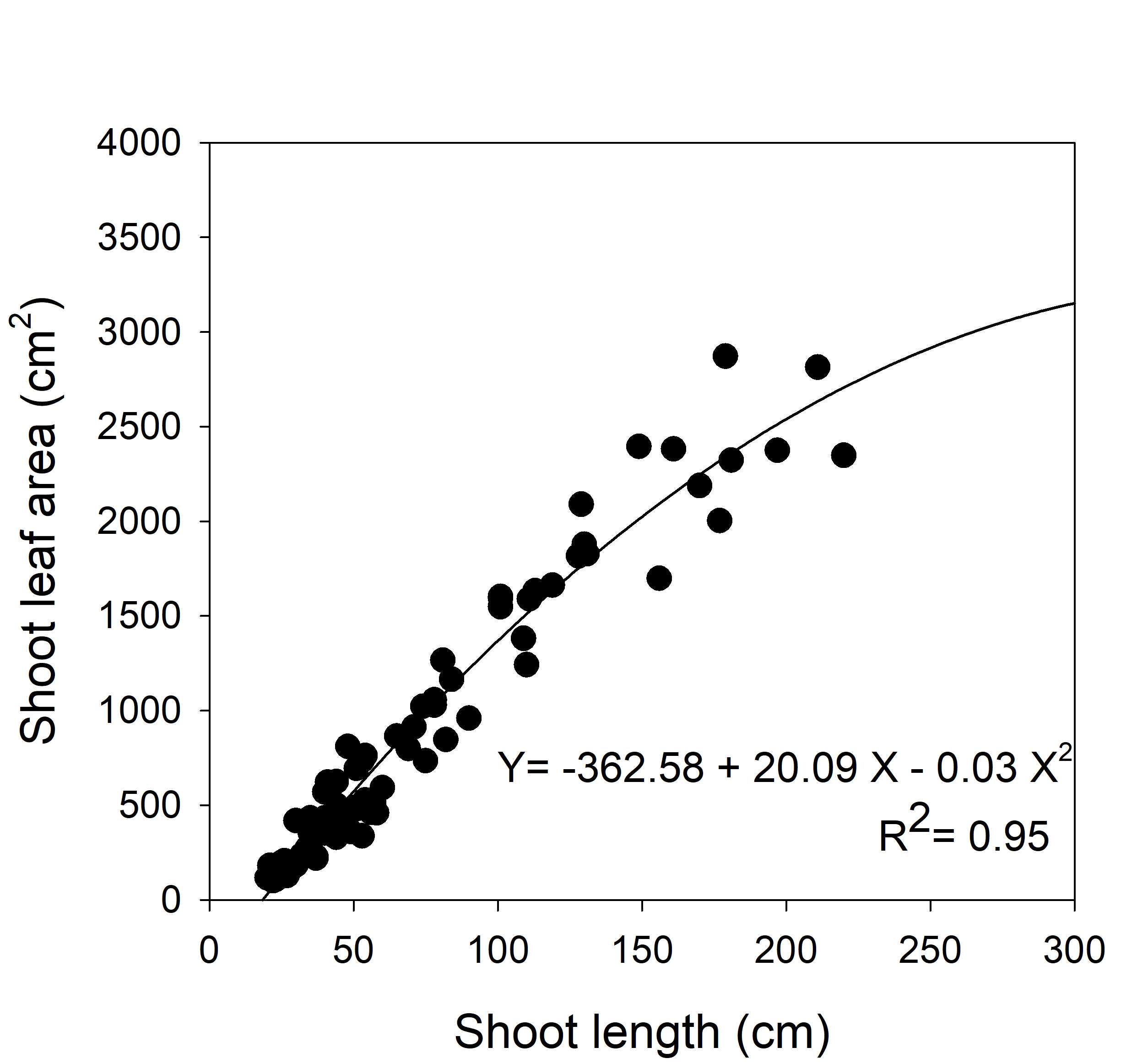

Supplement: Supplementary file 6 [file Image_6.JPEG]
